# Cervical Ectopic Pregnancy following Assisted Reproductive Technology: A Case Report

**Published:** 2012-12-17

**Authors:** Firoozeh Ahmadi, Shohreh Irani

**Affiliations:** 1 Department of Reproductive Imaging, Reproductive Biomedicine Research Center, Royan Institute for Reproductive Biomedicine, ACECR, Tehran, Iran; 2 Department of Epidemiology and Reproductive Health, Reproductive Epidemiology Research Center, Royan Institute for Reproductive Biomedicine, ACECR, Tehran, Iran

**Keywords:** Cervical Ectopic Pregnancy, Hemorrhage, ART

## Abstract

Cervical ectopic pregnancy (EP) is an infrequent, life-threatening form of ectopic gestation pregnancy that implants within the endocervical canal. With the increase in use of assisted reproductive technology (ART) worldwide and more liberal use of transvaginal sonography (TVS) during early pregnancy, more cases of cervical ectopic pregnancy are being diagnosed. Early diagnosis of this condition by using ultrasound imaging allows for prevention of maternal morbidity due to hemorrhage and leads to conservative management of this condition.We present the case of a 38-year old woman (gravida 1, para 0) who was found to have acervical ectopic pregnancy at six weeks of gestation.

## Introduction

Cervical pregnancy is a rare, dangerous type of ectopic pregnancy (EP) characterized by implantation of a fertilized ovum in the endocervical canal under the internal os level. Its frequency is less than 1% of all ectopic pregnancies (1 -3 ). Although cervical pregnancy has the potential for high morbidity due to massive hemorrhage; the mortality rate is low due to early ultrasonographic diagnosis. Thus diagnosing the condition as early as possible is of great importance (1 , 4 , 5 ). In this report, we describe the diagnosis and management of a case of cervical pregnancy at six weeks gestation.

### Case Report

**Fig 1:**
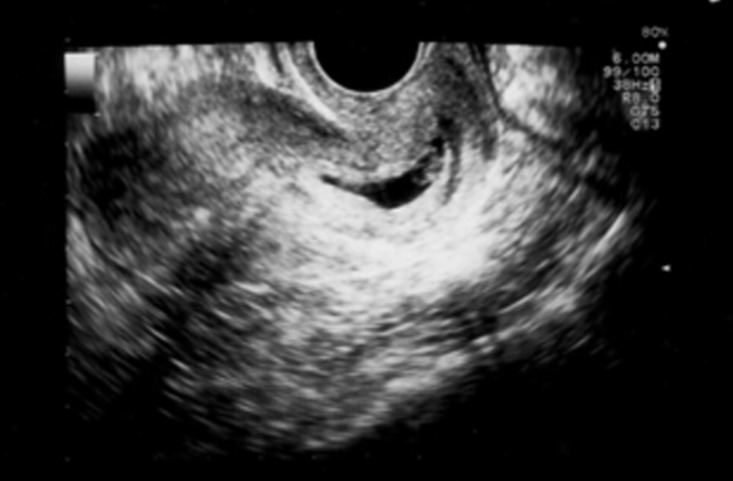
Sagittal endovaginal ultrasound of a cervical EP.

**Fig 2:**
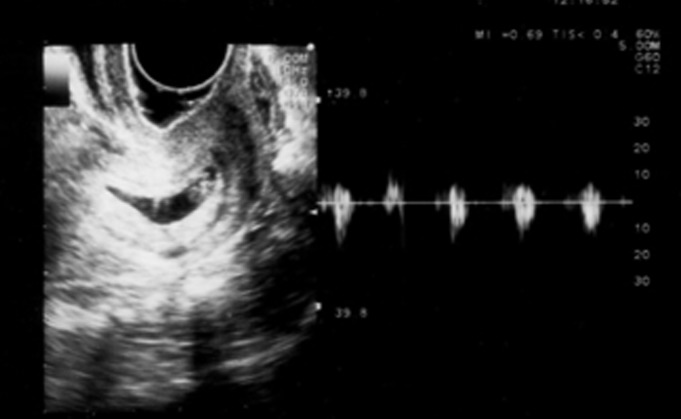
Ultrasound shows a seven-week gestation embryo with cardiac activity in the cervical canal

The patient was a 38-year-old female (gravida 1, para 0) with a 20-year history of infertility. In 2009 she underwent a hysteroscopy with resection of a broad, short septum. She became pregnant during the fourth intra-cytoplasmic sperm injection (ICSI) treatment at Royan Institute. The patient presented with a significantly elevated beta human choronic gonadotropin (beta hCG) level of >100 mIU/mL two weeks after embryo transfer (ET). Four weeks later, the beta hCG level was 1630 IU/mL. The first transvaginal ultrasound at 6.5 weeks gestational age showed an embryo, 5 mm in length with cardiac activity that was implanted in the cervical canal. No intrauterine pregnancy was seen. Since the pregnancy was so important for the mother, she was followed with repeated ultrasound studies. One week later, ultrasound confirmed a seven-week gestational age embryo with cardiac activity in the cervical canal (Figs 1, 2). She was referred to the hospital for medical treatment. Initially, 1cc of 10% KCl was injected in the gestational sac, followed by injection of 5cc methotrexate (50 mg) in the gestational sac and its surroundings. After two intramuscular methotrexate injection the β-human chorionic gonadotropin (β HCG) decreased. The complete disappearance of the gestational sac was confirmed by a follow-up ultrasound and the level of beta hCG immediately reduced during the weekly follow-ups (<5 mIU/mL).

## Discussion

The precise etiology of cervical pregnancy is unknown, although there are several factors which increase the incidence of cervical EP, such as endometrial damage following curettage, chronic endometritis, pelvic inflammatory disease (PID) (1 , 4 , 6 ), myoma, intrauterine devices (IUD), *in vitro* fertilization, and an anomaly of the embryo (1 , 4 ). The awareness of cervical ectopic pregnancy and its sonographic appearance is of great importance because it can be easily mistaken for other pathologies, such as heterotopic pregnancy, incomplete abortion, and normal pregnancy with low uterine implantation, EP in a cesarean section scar, nabotian cyst, and cervical mass. Due to the differences in treatment and practical management of these conditions, an accurate diagnosis is essential (3 , 6 ).

The absence of a gestational sac in the uterine cavity and the presenceof a gestational sac in the cervical canal are required for a diagnosis of cervical ectopic pregnancy (7 ). Cardiac activity is basically pathognomonic. The gestational sac in the cervix is typically eccentrically located and is either round or oval (6 ).

**Fig 3:**
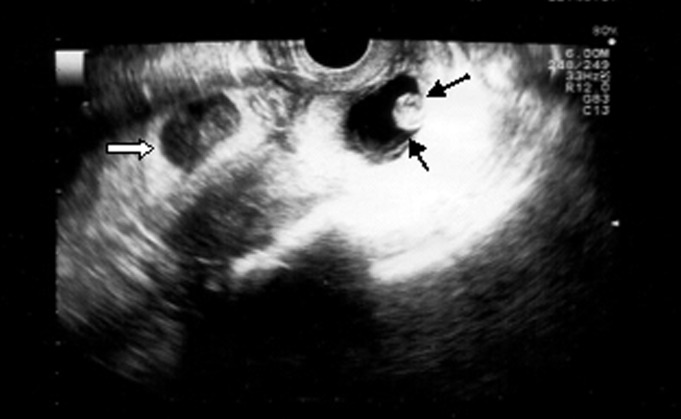
Two sacs simulating heterotopic pregnancy, one inside the uterus(white arrow) and the other in an ectopic site in the cervix (black arrows).

Distinguishing between a cervical ectopic pregnancy and a heterotopic pregnancy is important. Heterotopic pregnancy remains a diagnostic challenge .The coincidence of an intrauterine pregnancy with a cervical EP may be cause missing diagnosis of either intrauterine pregnancy or cervical EP. Recently there are more reports of heterotopic pregnancies, which most likely result from an increased use of ART and hormone therapy (3 , 6 , 8 ).We have presented another case of heterotopic pregnancy after ART in this center. As seen in figure 3, there are two fertilized eggs, one inside the uterus and the other in the ectopic site in the cervix.

Transvaginal three-dimensional ultrasound and color Doppler are useful as complementary imaging methods in order to correctly diagnose a cervical pregnancy by specifying the correct location of the gestational sac and showing the trophoblastic flow around the cervical sac (1 , 7 , 9 ).

The best way to diagnose ectopic cervical pregnancy is to observe a pregnancy inside the cervix stroma with a viable embryo. The cervical sac is usually round and similar to a normal pregnancy. It may, however, become elliptical or flattened, thus making diagnosis difficult (6 ). The internal Os is closed and there is a decidual reaction in the endometrium. The sac appears eccentric in the sonography and an hourglass appearance is seen due to implantation of the sac and dilation of the cervix (6 , 8 ).

In a spontaneous abortion, an irregular and deformed sac is seen without any surrounding echogenic ring and fetal heart activity. Differential diagnosis of cervical EP with the transit products of conception in spontaneous abortion was also made by gentle pressure of vaginal probe. In the spontaneous abortion, transit products of conception will be mobile which is called sliding sign (8 ).

In a repeat ultrasound a few hours later, we can possibly see the complete passage or movement of the gestational sac through the cervix. The uterus is large and the typical hourglass view of a cervical ectopic pregnancy is not visualized. The internal Os is open and the external Os may be opened or closed. β hCG levels are significantly decreased if it correlates with a miscarriage (6 , 3 , 8 ).

Several features have been suggested to help distinguish ectopic pregnancy in a cesarean section scar from cervical ectopic pregnancy. There is always a history of at least one prior cesarean delivery and generally a very thin myometrium can be seen in proximity to the bladder wall and region of the scar. In this type of ectopic pregnancy the trophoblastic tissue may also attack the bladder (3 , 6 , 10 ).

Late diagnosis of cervical EP can lead to a massive hemorrhage and, in the past, this complication frequently led to a hysterectomy. High risk of hemorrage is due to predominant amount of fibrous tissue in the cervix that lies at the edge of the small and thin layer of muscle in the cervix. Now conservative treatments are available, including sonographically-guided local potassium chloride injection, systemic or local methotrexate, and preoperative uterine artery embolization prior to dilatation and evacuation (3 , 6 , 11 , 12 ).

While cervical Ep is still uncommon, recently more cases have been reported may be due to increasing use of ART and routine transvaginal ultrasound scanning during pregnancy which leads to more detection of cervical EP (1 ).
